# Phylogenetic relationships in the genus *Avena* based on the nuclear *Pgk1* gene

**DOI:** 10.1371/journal.pone.0200047

**Published:** 2018-11-08

**Authors:** Yuanying Peng, Pingping Zhou, Jun Zhao, Junzhuo Li, Shikui Lai, Nicholas A. Tinker, Shu Liao, Honghai Yan

**Affiliations:** 1 Triticeae Research Institute, Sichuan Agricultural University, Chengdu, People’s Republic of China; 2 Collaborative Innovation Center of Tissue Repair Material of Sichuan Province, China West Normal University, Nanchong, People’s Republic of China; 3 Ottawa Research and Development Centre, Agriculture and Agri-Food Canada, Ottawa, Canada; Brigham Young University, UNITED STATES

## Abstract

The phylogenetic relationships among 76 *Avena* taxa, representing 14 diploids, eight tetraploids, and four hexaploids were investigated by using the nuclear plastid 3-phosphoglycerate kinase gene (*Pgk1*). A significant deletion (131 bp) was detected in all the C genome homoeologues which reconfirmed a major structural divergence between the A and C genomes. Phylogenetic analysis indicated the C_p_ genome is more closely related to the polyploid species than is the C_v_ genome. Two haplotypes of *Pgk1* gene were obtained from most of the AB genome tetraploids. Both types of the *barbata* group showed a close relationship with the A_s_ genome diploid species, supporting the hypothesis that both the A and B genomes are derived from an A_s_ genome. Two haplotypes were also detected in *A*. *agadiriana*, which showed close relationships with the A_s_ genome diploid and the A_c_ genome diploid, respectively, emphasizing the important role of the A_c_ genome in the evolution of *A*. *agadiriana*. Three homoeologues of the *Pgk1* gene were detected in five hexaploid accessions. The homoeologues that might represent the D genome were tightly clustered with the tetraploids *A*. *maroccana* and *A*. *murphyi*, but did not show a close relationship with any extant diploid species.

## Introduction

The genus *Avena* L. belongs to the tribe Aveneae of the grass family (Poaceae). It contains approximately 30 species [1–4] reflecting a wide range of morphological and ecological diversity over the temperate and subtropical regions [5]. The evolutionary history of *Avena* species has been discussed for decades, and remains a matter of debate despite considerable research effort in this field. Cytologically, three ploidy levels are recognized in the genus *Avena*: diploid, tetraploid, and hexaploid, with a base number of seven chromosomes [6, 7]. The diploids are divided clearly into two distinct lineages with the A and C genomes. All hexaploid species share the same genomic constitution of ACD, corroborated by fertile interspecific crosses among each other, as well as by their similar genome sizes [8]. With less certainty, the tetraploids have been designated as AB or AA, AC or DC, and CC genomes [9]. It is noteworthy that the B and D genomes within the polyploid species have not been identified in any extant diploid species. There are three C genome diploid species, which have been grouped into two genome types (C_p_ and C_v_) according to their karyotypes [10]. Both types show a high degree of chromosome affinity to the polyploid C genome [9–14], but none have been undisputedly identified as the C genome progenitor of the polyploids.

The A genome origin of polyploid oats has also been under intense scrutiny. However, there is no conclusive evidence regarding which the A genome diploid contributed to the polyploid oats. There are up to 12 species designated as A genome diploids. These species have been further subdivided into five sub-types of A_c_, A_d_, A_l_, A_p_ and A_s_ genomes, according to their karyotypes [6, 7]. Most research based on karyotype comparisons [6, 15], in situ hybridization [11, 16–18], as well as the alignments of nuclear genes [13, 14] suggest that one of the A_s_ genome species may be the A genome donor of polyploid oats. Alternatively, some studies have proposed the A_c_ genome diploid *A*. *canariensis* [19], or the A_l_ genome diploid *A*. *longiglumis* [9, 12] as the most likely A genome donor.

The absence of diploids with the B and D genomes complicates the B and D genome donor identification. It is generally accepted that both B and D genomes are derived from A genomes, due to the high homology between the B and A genomes [11, 20], as well as between the D and A genomes [16, 19, 21]. Our recent study based on high-density genotyping-by-sequencing (GBS) markers [9] provided strong evidence that the three tetraploid species formerly designated as AC genomes are much closer to the C and D genomes of the hexaploids than they are to the hexaploid A genome. These findings suggest that the hexaploid D genome exists in the extant tetraploids. However, no extant diploid species, even the A_c_ genome diploid *A*. *canariensis*, which was considered as the most likely D genome progenitor based on direct evidence from morphological features [22] and indirect evidence from fluorescent in situ hybridization (FISH) [18], showed enough similarity to the D genome of tetraploid and hexaploid oats to warrant consideration as a direct D genome progenitor.

In the case of the B genome, an initial study of chromosome pairing of hybrids between the AB genome tetraploids and the A_s_ genome diploids suggested that the B genome arose from the A_s_ genome through autoploidization [23]. This hypothesis was supported by another GBS study [19], which showed that the AB genome tetraploid species fell into a tight cluster with A_s_ genome diploids. However, other evidence from C-banding [24], FISH [17], RAPD markers [25], and DNA sequence alignment [14] has indicated a clear distinction between A and B genomes, suggesting an allotetraploid origin of the AB genome tetraploid species. The most probable A genome progenitor of the AB genome tetraploids is assumed to be an A_s_ genome diploid species, while the B genome of these species remains controversial.

Single or low copy nuclear genes are widely used in phylogenetic analyses due to their bi-parental inheritance and to the informativeness of mutations. Such studies have successfully revealed multiple polyploid origins, and clarified hybridization events in a variety of plant families [26, 27]. In a previous study [14], we investigated the relationships among *Avena* species by sequencing the single-copy nuclear Acetyl-CoA carboxylase gene (*Acc1*). The results provided some useful clues to the relationships of *Avena* species.

The *Pgk1* gene, which encodes the plastid 3-phosphoglyceratekinase, is another nuclear gene that has been widely used to reveal the evolutionary history of the *Triticum/Aegilops* complex due to its single copy status per diploid chromosome in grass [26, 28, 29]. The *Pgk1* gene is now considered to be superior to the *Acc1* gene in phylogenetic analysis, since it has more parsimony informative sites than the *Acc1* gene [26, 29]. In the present study, we sequenced cloned *Pgk1* gene copies from 76 accessions representing the majority of *Avena* species, in an attempt to further clarify evolutionary events in this important genus.

## Materials and methods

### Plant materials

A total of 76 accessions from26 *Avena* species were investigated to represent the geographic range of six sections in *Avena*, together with one accession from *Trisetopsis turgidula* as a functional outgroup (Table 1). All seeds were provided by Plant Gene Resources of Canada (PGRC) or the National Small Grains Collection, Agriculture Research Service, United States Department of Agriculture (USDA, ARS) with the exception of the three accessions of *A*. *insularis*, which were kindly provided by Dr. Rick Jellen, Brigham Young University, Provo, UT, USA. The species *A*. *atherantha*, *A*. *hybrida*, *A*. *matritensis* and *A*. *trichophylla* described in Baum’s [1] monograph and *A*. *prostrata* described by Ladizinsky [30] were not included due to a lack of viable material.

**Table 1:** List of materials used in the present study including species, haplomes, accession number, origin, the number of sequenced clones, abbreviation displayed in MJ network, and the sequence number in Genbank (https://www.ncbi.nlm.nih.gov).

Taxa	Haplomes	Accession Number	Origin*	Number of sequenced clones	Abbrev- iation	Genbank Accession
Section *Ventricosa*						
*A*. *clauda* Dur.	C_p_	CN 19242	Turkey	6	CLA1_1	KU888786
		CN 21378	Greece	2	CLA2_1	KU888787
		CN 21388	Algeria	2	CLA3_1	KU888804
		CN 24695	Turkey	2	CLA4_1	KU888784
*A*. *eriantha* Dur. (syn *A*. *pilosa* Bieb.)	C_p_	CIav 9050	United Kingdom	2	ERI1_1	KU888785
		PI 367381	Madrid, Spain	2	ERI2_1	KU888805
*A*. *ventricosa* Balansa ex Coss.	C_v_	CN 21405	Algeria	2	VEN1_1	KU888806
		CN 39706	Azerbaijan	2	VEN2_1	KU888807
Section *Agraria*						
*A*. *brevis* Roth	A_s_	CIav 1783	German	1	BRE1_1	KU888707
		CIav 9113	Europe	2	BRE2_1	KU888718
		PI 258545	Portugal	1	BRE3_1	KU888710
*A*. *hispanica* Ard.	A_s_	CN 25676	Portugal	2	HIS1_1	KU888714
		CN 25727	Portugal	2	HIS2_1	KU888711
		CN 25766	Portugal	2	HIS3_1	KU888709
		CN 25778	Portugal	1	HIS4_1	KU888712
*A*. *nuda* L.	A_s_	PI 401795	Netherlands	2	NUD1_1	KU888734
*A*. *strigosa* Schreb.	A_s_	PI 83722	Australia	6	STR1_1	KU888719
		PI 158246	Lugo, Spain	2	STR2_1	KU888713
		CIav 9066	Ontario, Canada	3	STR3_1	KU888708
Section *Tenuicarpa*						
*A*. *agadiriana* Baum & Fedak	AB	CN 25837	Africa: Morocco	5	AGA1_1	KU888753
					AGA1_2	KU888774
		CN 25854	Africa: Morocco	4	AGA2_1	KU888777
					AGA2_2	KU888754
		CN 25856	Africa: Morocco	3	AGA3_1	KU888776
					AGA3_2	KU888751
		CN 25863	Africa: Morocco	3	AGA4_1	KU888775
		CN 25869	Africa: Morocco	4	AGA5_1	KU888752
					AGA5_2	KU888778
*A*. *atlantica* Baum & Fedak	A_s_	CN 25849	Africa: Morocco	2	ATL1_1	KU888757
		CN 25859	Africa: Morocco	1	ATL2_1	KU888756
		CN 25864	Africa: Morocco	2	ATL3_1	KU888739
		CN 25887	Africa: Morocco	2	ATL4_1	KU888737
		CN 25897	Africa: Morocco	1	ATL5_1	KU888736
*A*. *barbata* Pott ex Link	AB	PI 296229	Northern, Israel	5	BAR1_1	KU888723
		PI 337802	Izmir, Turkey	8	BAR2_1	KU888722
					BAR2_2	KU888732
		PI 337826	Greece	6	BAR3_1	KU888720
		PI 282723	Northern, Israel	6	BAR4_1	KU888729
		PI 337731	Macedonia, Greece	8	BAR5_1	KU888731
		PI 367322	Beja, Portugal	6	BAR6_1	KU888730
*A*. *canariensis* Baum et al	A_c_	CN 23017	Canary Islands	6	CAN1_1	KU888779
		CN 23029	Canary Islands	2	CAN2_1	KU888782
		CN 25442	Canary Islands	1	CAN3_1	KU888780
		CN 26172	Canary Islands	2	CAN4_1	KU888783
		CN 26195	Canary Islands	2	CAN5_1	KU888781
*A*. *damascena* Rajah & Baum	A_d_	CN 19457	Syria	1	DAM1_1	KU888744
		CN 19458	Syria	2	DAM2_1	KU888745
		CN 19459	Syria	2	DAM3_1	KU888747
*A*. *hirtula* Lag.	A_s_	CN 19530	Antalya, Turkey	2	HIR1_1	KU888738
		CN 19739	Algeria	2	HIR2_1	KU888762
		CN 21703	Morocco	2	HIR3_1	KU888717
*A*. *longiglumis* Dur.	A_l_	CIav 9087	Oran, Algeria	6	LON1_1	KU888741
		CIav 9089	Libya	2	LON2_1	KU888749
		PI 367389	Setubal, Portugal	1	LON3_1	KU888750
*A*. *lusitanica* Baum	A_s_	CN 25885	Morocco	1	LUS1_1	KU888746
		CN 25899	Morocco	1	LUS2_1	KU888748
		CN 26265	Portugal	2	LUS3_1	KU888742
		CN 26441	Spain	2	LUS4_1	KU888763
*A*. *wiestii* Steud.	A_s_	PI 53626	Giza, Egypt	2	WIE1_1	KU888715
		CIav 9053	Ontario, Canada	2	WIE2_1	KU888716
Section *Ethiopica*						
*A*. *abyssinica* Hochst.	AB	PI 411163	Seraye, Eritrea	4	ABY1_1	KU888724
		PI 411173	Tigre, Ethiopia	6	ABY2_1	KU888740
					ABY2_2	KU888725
*A*. *vaviloviana* Mordv.	AB	PI 412761	Eritrea	4	VAV1_1	KU888743
					VAV1_2	KU888728
		PI 412766	Shewa, Ethiopia	5	VAV2_1	KU888726
					VAV2_2	KU888735
Section *Pachycarpa*						
*A*. *insularis* Ladiz.	AC(DC)	sn	Sicily, Italy	4	INS1_1	KU888794
					INS1_2	KU888705
		6-B-22	Sicily, Gela, Italy	4	INS2_1	KU888706
					INS2_2	KU888796
		INS-4	Sicily, Gela, Italy	3	INS3_1	KU888790
					INS3_2	KU888704
*A*. *maroccana* Grand. (syn. *A magna* Murphy et Terrell)	AC(DC)	CIav 8330	Morocco	3	MAR1_1	KU888773
					MAR1_2	KU888799
		CIav 8331	Khemisset, Morocco	3	MAR2_1	KU888721
					MAR2_2	KU888800
*A*. *murphyi* Ladiz.	AC(DC)	CN 21989	Spain	4	MUR1_1	KU888767
					MUR1_2	KU888802
		CN 25974	Morocco	3	MUR2_1	KU888769
					MUR2_2	KU888788
Section *Avena*						
*A*.*fatua* L.	ACD	PI 447299	Gansu, China	6	FAT1_1	KU888768
					FAT1_2	KU888795
					FAT1_3	MH780169
		PI 544659	United States	7	FAT2_1	KU888764
					FAT2_2	KU888760
					FAT2_3	KU888798
*A*.*occidentalis* Dur.	ACD	CN 4547	Canary Islands, Spain	6	OCC1_1	KU888791
					OCC1_2	MH780167
					OCC1_3	MH780165
		CN 23036	Canary Islands, Spain	8	OCC2_1	KU888755
					OCC2_2	KU888803
					OCC2_3	KU888771
		CN 25942	Morocco	7	OCC3_1	KU888733
					OCC3_2	KU888789
					OCC3_3	KU888758
		CN 25956	Morocco	8	OCC4_1	KU888801
					OCC4_2	KU888772
*A*. *sativa* L.	ACD	PI 194896	Gonder, Ethiopia	6	SAT1_1	KU888727
					SAT1_2	KU888759
					SAT1_3	KU888793
		PI 258655	Russian Federation	8	SAT2_1	KU888797
					SAT2_2	KU888766
					SAT2_3	KU888761
*A*. *sterilis* L.	ACD	PI 411503	Alger, Algeria	8	STE1_1	KU888765
					STE1_2	MH780168
		PI 411656	Tigre, Ethiopia	7	STE2_1	KU888792
					STE2_2	KU888770
					STE2_3	MH780166
Outgroup						
*Trisetopsis turgidula* Röser & A. Wölk		PI 364343	Maseru, Lesotho	1		KU888808

* Origin represents the collection site of wild material where this information is available, otherwise it represents the earliest source for which information is available.

### DNA isolation, cloning and sequencing

Genomic DNA was isolated from fresh leaves of single plants following a standard CTAB protocol [31]. *Pgk1* gene sequences were amplified by using a pair of *Pgk1*-specific primers, PGKF1 (5’-TCGTCCTAAGGGTGTTACTCCTAA-3’) and PGKR1 (5’-ACCACCAGTTGAGATGTGGCTCAT-3’) described by Huang et al. [28]. Polymerase chain reactions (PCR) were carried out under cycling conditions reported previously [26]. High fidelity *Taq* DNA polymerase (*Ex*-*Taq*, Takara, Japan, Cat^#^RR001A) was used to reduce the potential PCR-based mutation. After estimating the size by 1.0% agarose gel, PCR products were purified using the QIAquick gel extraction kit (QIAGEN Inc., USA). The purified products were cloned into the pMD19-T vector (Takara) following the manufacturer's instructions. Initially, 6–8 positive clones from each of four accessions from 4 diploid species, including *A*. *canariensis* (A_c_), *A*. *longiglumis* (A_l_), *A*. *strigosa* (A_s_), and *A*. *clauda* (C_p_), were sequenced to confirm that the *Pgk1* gene was present in *Avena* diploid species as a single copy. After confirming its single copy status in diploid species, 2–3 positive clones were selected and sequenced from each accession of the remaining diploid species. In order to isolate all possible homoeologous sequences in polyploid species, 4–6 positive clones from each accession of the tetraploid species and 5–10 positive clones from each accession of the hexaploid species were selected and sequenced. All the cloned PCR products were sequenced on both strands by a commercial company (Sangon Biotech Co., Ltd., Shanghai, China) based on Sanger sequencing technology.

### Sequence alignment and phylogenetic analysis

The homology of sequences was verified usingthe BLAST program in NCBI. In order to reduce the matrix size of the dataset, redundant sequences were removed, keeping one representative sequence if several identical sequences were derived from the same accession. Sequences were aligned using ClustalW software with default parameters [32] followed by manual correction. Substitution saturation of *Pgk1* sequences was examined using DAMBE version 5 [33] by calculating and plotting pairwise rates of transitions and transversions against sequence divergence under the TN93 model. Phylogenetic trees were created by using maximum parsimony (MP), and Bayesian inference (BI). MP analysis was performed on PAUP* 4.0b10 [34] using the heuristic search with 100 random addition sequence replicates and Tree Bisection-Reconnection (TBR) branch swapping algorithms. Bootstrapping with 1000 replicates was estimated to determine the robustness of formed branches [35]. Gaps in the sequence alignment were disregarded using the option “gapmode = missing”, which is consistent with an assumption that insertion/deletion events are an independent stochastic process from SNP substitutions. BI analysis was carried out by using MrBayes v3.2 [36]. The best-fit substitution model for BI analysis was GTR+Γ+I, which was determined by using MrModelTest v2.3 under Akaike information criteria (AIC) (http://www.ebc.uu.se/systzoo/staff/nylander.html). Four Markov chain Monte Carlo (MCMC) chains with default priors settings were run simultaneously. To ensure the two runs converged onto the stationary distribution, 6,000,000 generations were run to make the standard deviation of split frequencies fall below 0.01. Samples were taken every 100 generations. The first 25% samples from each run were discarded as the “burn-in”. The 50% majority-rule consensus tree was constructed from the remaining trees. Posterior probability (PP) values were used to evaluate the statistical confidence of each node.

### Network analysis

The median-joining (MJ) network [37] method has been demonstrated to be an effective method for assessing the relationship in closely related lineages [38], and thus was applied in this study. As MJ algorithms are designed for non-recombining molecules [37], DNA recombination was test by using a pragmatic approach-Genetic Algorithm Recombination Detection (GARD), described by Pond et al. [39]. The test was carried out on a web-based interface for GARD at http://www.datamonkey.org/GARD/. Building upon this test, the intron data was used for MJ reconstruction due to the absence of recombination signal, while potential recombination signals were detected in the exon regions. The MJ network analyses was performed using the Network 4.6.1.4 program (Fluxus Technology Ltd, Clare, Suffolk, UK).

## Results

### Sequence analysis

A total of 268 clones were sequenced from 76 accessions of 26 *Avena* species. BLASTn analysis indicated that these sequences ranged in identity from 84% to 87% with wheat *Pgk1* (AF343478) with high query coverage (more than 90%), and from 77% to 100% with wheat *Pgk2* (AF343449) but with very low query coverage (less than 35%), confirming the proper identity of all clones as *Pgk1*. Following removal of the redundant sequences within each accession, 109 sequences were identified, including one from each of the 44 diploid accessions, 37 unique sequences from 22 tetraploids, and 28 from 10 hexaploids. Theoretically, 44 homoeologues should be isolated from 22 tetraploid accessions, and 30 single-copy homoeologues were expected from 10 hexaploid accessions. Despite a high number of cloning attempts in *A*. *barbata* accession (Table 1), only one copy was detected in five of its six accessions. Whereas two very similar (only one site varied in exon 2) copies were detected in the sixth accession. It is possible that these accessions contain genomes of high similarity or autopolyploid origin. Another possibility that cannot be ruled out within the polyploids is the loss of one gene copy through homoeologous recombination or deletion.

**Fig 1:**
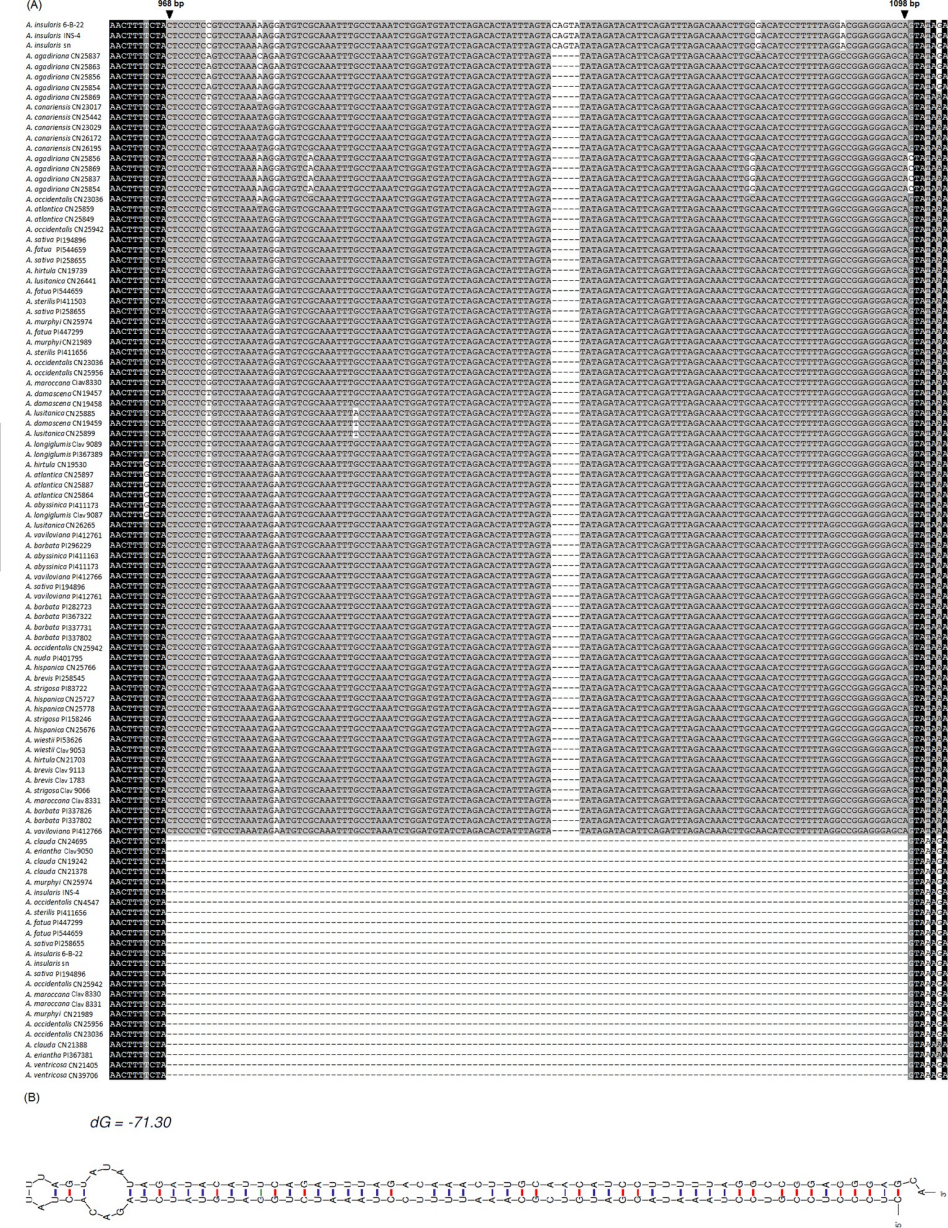
*Pgk1* gene sequence analysis. (A) Partial alignment of the amplified *Pgk1* gene of *Avena* species (B) Secondary structure of the deletion sequence between the A and C genomes.

All of the *Pgk1* gene sequences isolated in this study contain 5 exons and 4 introns, covering a total length from 1391 bp to 1527 bp, which is consistent with previous studies of this gene in wheat [28] and *Kengyilia* [26]. The alignment of *Pgk1* sequences was edited and deposited in TreeBase (http://treebase.org) under following URL: http://purl.org/phylo/treebase/phylows/study/TB2:S23228. Including both exons and introns, this alignment resulted in a matrix of 1539 nucleotide positions, of which 11.6% (179/1539) were variable, and 10.1% (156/1539) were parsimony informative. The nucleotide frequencies were 0.259 (A), 0.300 (T), 0.206 (C), and 0.235 (G). A significant (131 bp) insertion/deletion feature (Fig 1A) occurred at position 968, whereby all non-C genome type sequences contained the inserted (or non-deleted) region. Further analysis indicated that this region is likely an inserted inverted repeat, which belongs to the MITE stowaway element. Its secondary structure is shown in Fig 1B. This insertion/deletion event could be used as a genetic marker for rapid diagnosis of *Avena* species containing the C genome.

### Phylogenetic analyses

**Fig 2:**
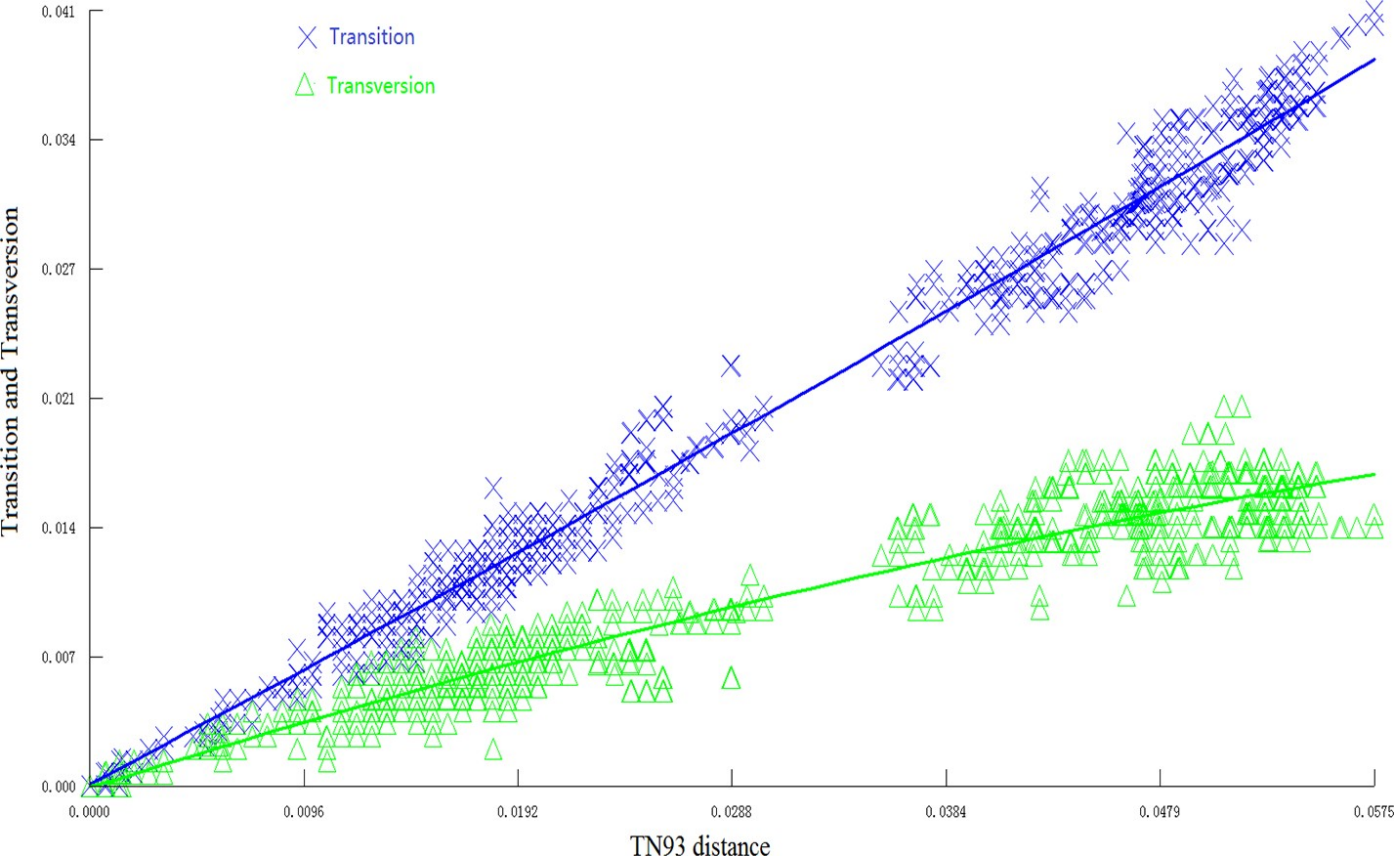
Saturation plot for transition and transversion of *Pgk1* gene sequences. The crosses are the number of transition events; the triangles are the number of transversion events. The x axis shows the genetic distance based on the TN93 model; the y axis is the proportion of transitions or tansversions, which was calculated by using the number of transitions or transversions divided by the sequence length. The curves show the trends of the variance of transitions and transversions with the genetic distance increasing.

The substitution plot for *Pgk1* (Fig 2) indicated that the *Pgk1* gene was not saturated and that it could be used for phylogenetic analysis. Phylogenetic trees of 76 *Avena* accessions with the oat-like species *Trisetopsis turgidula* as outgroup were generated through maximum parsimony and Bayesian inference approaches on the non-redundant dataset. The parsimony analysis resulted in 80 equally parsimonious trees (consistency index (CI) = 0.637, retention index (RI) = 0.956). BI analysis inferred an almost identical tree topology as the MP analysis (S1 Fig).

**Fig 3:**
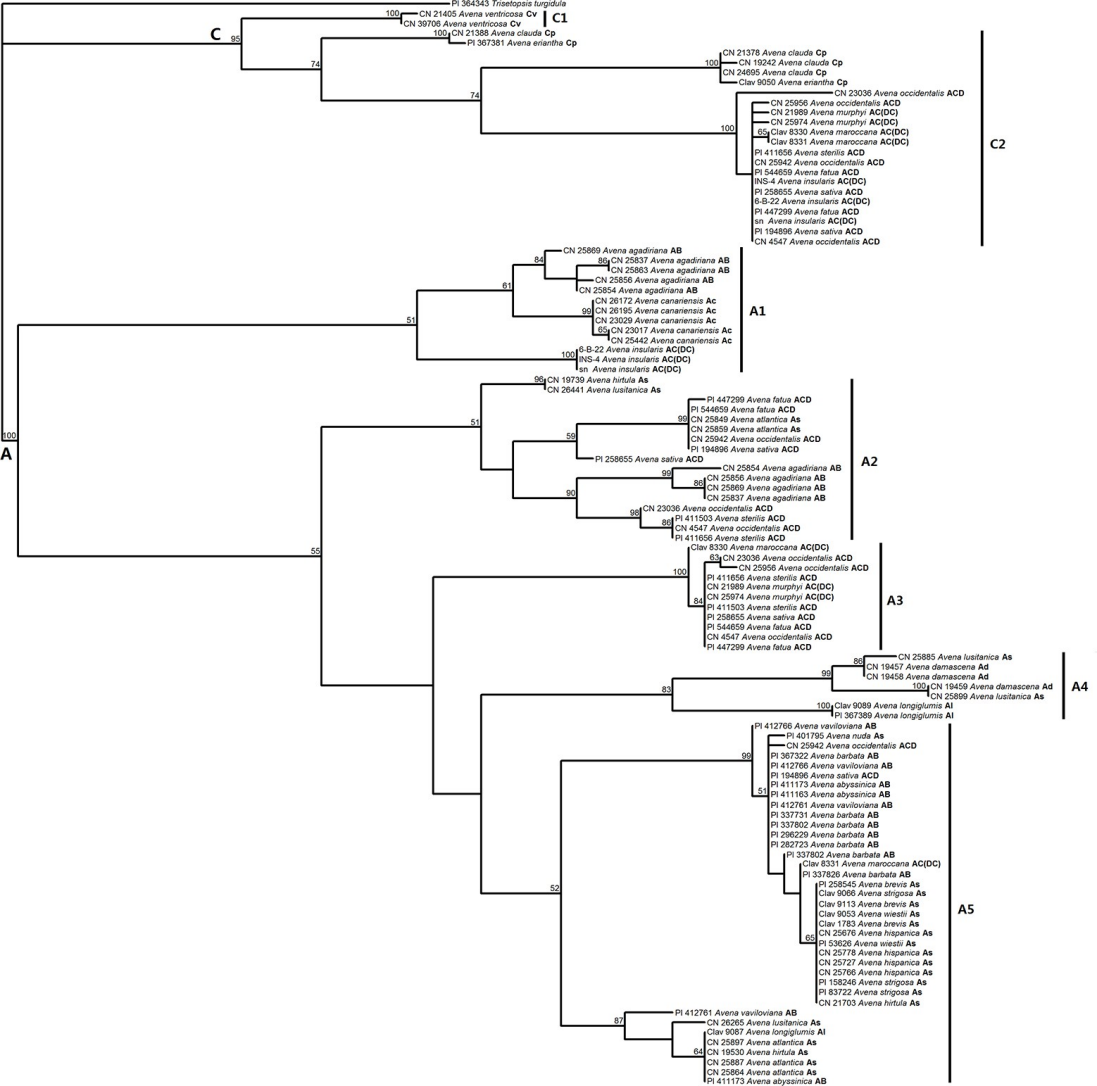
Maximum parsimony tree derived from *Pgk1* sequence data. The tree was constructed using a heuristic search with TBR branch swapping. Numbers above the branches are bootstrap support (BS) values ≥50%. Accession number, species name and haplome are indicated for each taxon.

Both Fig 3 and S1 Fig show that the *Pgk1* gene sequences from 76 *Avena* accessions were split into two distinct clades with high BS (100% and 95%) and PP (100% and 100%) support. One clade contained all C-genome type sequences, hence referred to as the C genome clade. The other clade contained all sequences from the species carrying the A genome, henceforth, referred to as the A genome clade. The C genome clade was composed of two major subclades. All C_v_ genome diploids formed the subclade C1 with 100% BS and 100% PP support, while subclade C2 included six C_p_ diploid accessions, seven AC(DC) genome tetraploid accessions and nine hexaploid accessions with 74% BS and 99% PP support. The *Pgk1* gene sequences in the A genome clade were further split into five major subclades. One genome copy of the AC(DC) genome tetraploid species *A*. *insularis* clustered with five accessions of the A_c_ genome diploid species *A*. *canariensis* and one genome homoeologue of the AB genome tetraploid species *A*. *agadiriana*, consequently forming the subclade A1 with low BS (51%) and PP (less than 90%) support. Within A1, the A_c_ genome diploids showed close relationships with the AB genome tetraploid species *A*. *agadiriana*. Subclade A2 was composed of four accessions of the AB genome tetraploids *A*. *agadiriana*, nine hexaploid accessions (*A*. *occidentalis* CN 23036, CN 25942 and CN 4547, *A*. *sativa* PI 194896 and PI 258655, *A*. *fatua* PI 447299 and PI 544659, *A*. *sterilis* PI 411503 and PI 411656) and four A_s_ genome diploid accessions (*A*. *atlantica* CN25849 and CN 25859, *A*. *lusitanica* CN 26441, and *A*. *hirtula* CN 19739). One genome sequence of the AC(DC) genome tetraploids (without *A*. *insularis*) together with eight hexaploid taxa formed a homogeneous clade (A3) that was separated from other species with high BS (100%) and PP (100%) support. The subclade A4 consisted of the A_d_ genome diploid *A*. *damascena*, the A_l_ genome diploid *A*. *longiglumis*, and the A_s_ genome diploid *A*. *lusitanica*. The remaining sequences from the A genome diploids and the AB genome tetraploids (without *A*. *agadiriana*) formed a relatively broader cluster A5, together with two hexaploid accessions (*A*. *sativa* PI 194896 and *A*. *occidentalis* CN 25942) and one AC(DC) genome tetraploid accession (*A*. *maroccana* CIav 8831).

Three groups of haplotypes of *Pgk1* sequences were identified in eight of ten hexaploid accessions (*A*. *fatua* PI 447299 and PI 544659, *A*. *occidentalis* CN 25942, CN 23036, and CN 4547, *A*. *sativa* PI 194896 and PI 258655, and *A*. *sterilis* PI 411656). These sequences fell into four subclades. One group clustered with the C genome diploids in subclade C2, and one group clustered with AC(DC) genome tetraploids in subclade A3. We hypothesize that these two types represent homoeologues from the C and D genomes, respectively. This interpretation is consistent with strong evidence presented by Yan et al. [9] that the AC(DC) tetraploids contain the progenitor D genome of the hexaploids. A third and fourth group fell into subclades A2 and A5. Since these two groups are highly separated, it is possible that they represent different A-genome events leading to different hexaploid lineages.

### Network analysis

**Fig 4:**
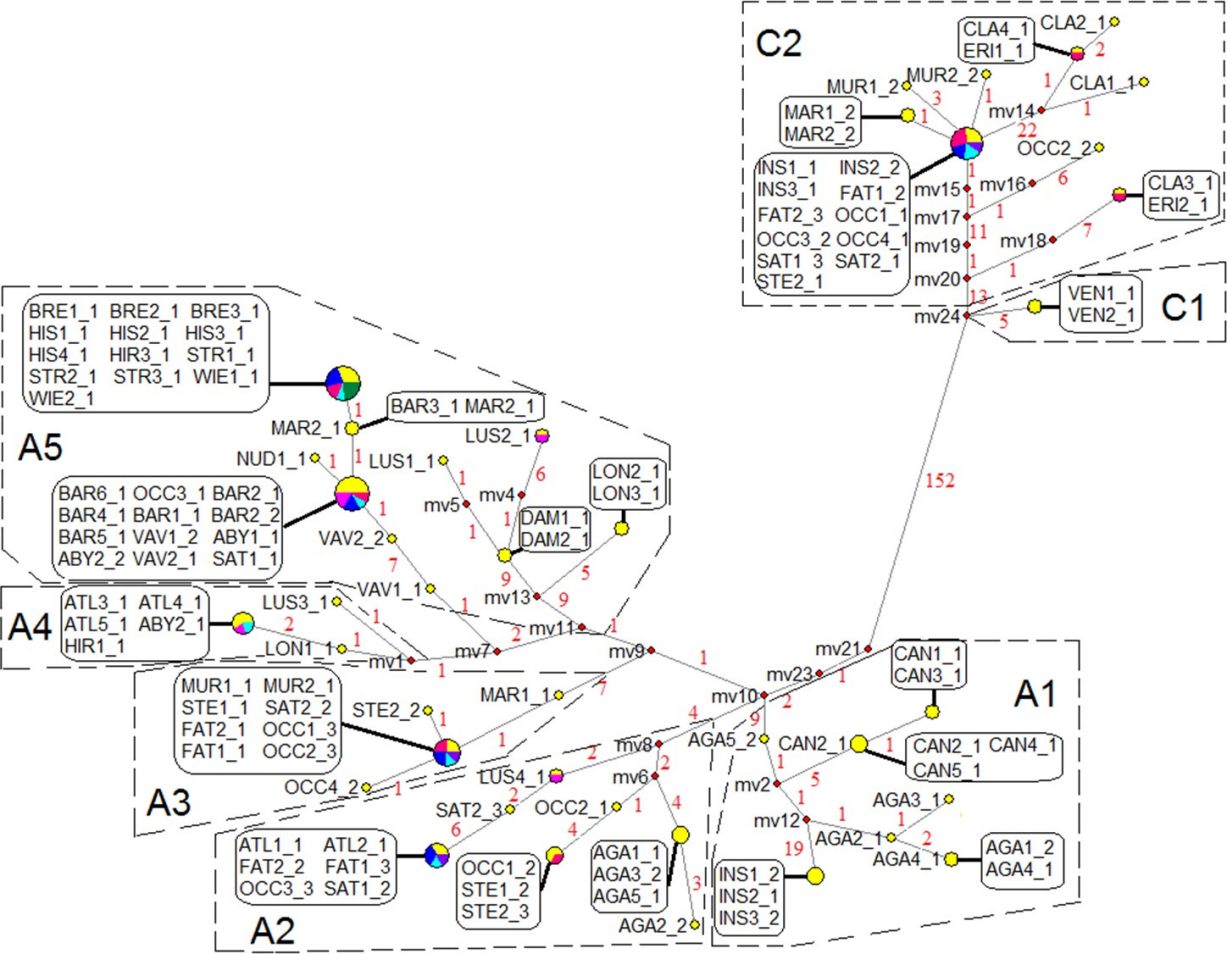
Median-joining networks based on 41 *Pgk1* gene haplotypes of intron regions derived from 26 *Avena* species. Each circular node represents a single haplotype, with relative size being proportional to the frequency of that haplotype. Distinct colors in the same haplotype node represent different species sharing the same haplotype (colors are arbitrary). Median vectors (mv) represent the putative missing intermediates. Numbers along network branches indicate the number of bases involved in mutations between two nodes. Clusters (surrounded by dashed lines) are named based on clade names shown in the MP tree (Fig 3). Three-letter abbreviations of species names are listed in Table 1. The numbers immediately after each species abbreviation represent different accessions of the same species, and the number following the underscore identifies different haplotypes from the same accession.

To gain better insight into relationships within closely related lineages, MJ network reconstruction based on the haplotypes of *Pgk1* sequences was employed. Due to the potential presence of recombination in the exon regions, the intron data was used for MJ network reconstruction. A total of 41 haplotypes were derived from 109 *Pgk1* gene sequences (Fig 4). This low level of haplotype diversity demonstrates the high conservation of this gene within genus *Avena*. The MJ network recovered a nearly identical phylogenetic reconstruction to that based on the MP and BI trees, therefore we identified the clades from the MP results (Fig 3) within the MJ network (Fig 4). Based on the topology and frequency of haplotypes, the MJ network was split into two main groups. The two major groups representing two distinct types of haplotypes (A and C genomes) were distinguished due to the 131 bp insertion/deletion. Ten C genome haplotypes were observed, which were much less diverse than the 31 A genome haplotypes. The two main groups were further subdivided into clusters corresponding to the seven MP-based subclades discussed earlier.

## Discussion

### Two distinct diploid lineages exist in genus *Avena*

A significant 131 bp insertion/deletion separated all *Avena* diploid species into two distinct groups representing the A and C genomes, respectively (Figs 1 and 4). These groups were also separated based on the MP or BI analysis that ignored gaps (Fig 3 and S1 Fig), indicating that the separation of A and C genomes is the most ancient major articulation in the genus *Avena*, a result that is consistent with most other literature [13, 14, 40]. MJ network analysis revealed that the C genome diploids have much lower levels of haplotype diversity than the A genome diploids. Within the C genome diploids, the C_p_ genome haplotypes were relatively more diverse than those of the C_v_ genome. These results might be explained by the geographic distribution of these species. The A genome diploids are distributed in a large region between latitude 20 and 40° N, while the C genome diploid species are restricted to a narrow territory along the Mediterranean shoreline [1]. The geographic distributions of the C genome diploid species are overlapping, but the range of the C_p_ genome diploid species is much broader than that of the C_v_ genome diploid species [41].

The A genome diploid species are the most diverse set of species in genus *Avena*, and chromosome rearrangements have occurred during the divergence of A-genomes from a common progenitor [41], resulting in the subdivision of the A genome into five types, of which we have investigated four. Our results showed that species with genome types A_c_, A_l_, and A_d_ formed groups that correspond well with previously reported structural differences. However, the A_s_ genome diploids appear to be much more diverse than previously reported, and are scattered into different subclades (Figs 3 and 4). Baum [1] divided all A_s_ genome diploids into two sections, section *Agraria* and section *Tenuicarpa*. All species of section *Agraria* have florets with a domesticated (non-shattering) base, whereas the other A_s_ species share relatively narrow spikelets. However, classification based on simple morphological traits is increasingly controversial. In this study, the A_s_ genome diploid species of section *Agraria* showed high degree of genetic homogeneity, consistently forming their own subclade A5, but other A_s_ genome species in section *Tenuicarpa* did not have their own subclade. *A*. *wiestii* showed a close relationship with the species of section *Agraria*, suggesting that it may be better-classified within that section. This result is in agreement with previous studies based on RAPD [42] and karyotypic comparisons [43]. Accessions of the other two A_s_ genome species of section *Tenuicarpa* (*A*. *atlantica* and *A*. *hirtula*) were scattered into different subclades. These results were also observed in other studies [13,14]. *A*. *lusitanica*, another A_s_ species of section *Tenuicarpa*, was diverged from other A_s_ species, but showed a close relationship to those with the A_d_ genome species *A*. *damascena*. This divergence has also been observed in many other studies [8, 9, 14, 40]. These, and other incongruences between morphological characters and genetic differences raise questions about appropriate taxonomical classifications among A_s_ genome species.

### The A_s_ and A_c_ genomes played roles in the AB tetraploid formation

Four recognized species have been proposed to have an AB genome composition. Of these, *A*. *barbata*, *A*. *abyssinica* and *A*. *vaviloviana* are grouped into a biological species known as the *barbata* group, while *A*. *agadiriana* is distinct [25]. Our results confirmed the reported structural differences between these two groups (Fig 3). Two different *Pgk1* gene sequences were detected from most of the AB genome tetraploids, supporting their allotetraploid origins. However, the genomes of *A*. *barbata* showed the least divergence, with only one of six *A*. *barbata* accessions providing multiple sequences, both of which were very similar. It seems that little divergence has occurred within the genome of *A*. *barbata* compared with that of *A*. *abyssinica* and *A*. *vaviloviana*. This result has also been observed in previous study based on FISH and southern hybridization analysis [17], which found some B chromosomes of *A*. *vaviloviana* are involved in inter-genomic translocations, while these rearrangements were not detected in *A*. *barbata*. There is little doubt that the A genome diploids have been involved in the formation of the *barbata* species. Some studies have suggested that both the A and B genomes of *barbata* species are diverged A_s_ genomes [16, 23, 44], while some others proposed that the B genome might have originated from other A genome diploid species [24, 25, 45]. In this study, both types of *Pgk1* sequences detected from the *barbata* group showed high degree of genetic homogeneity with the A_s_ genome diploids (Fig 3), thus it was impossible to determine which type represents the A or B genome.

The recently discovered tetraploid species *A*. *agadiriana* was also proposed to have an AB genome composition because of its high affinity with *A*. *barbata* [23]. However, this designation has been questioned due to chromosomal divergences between *A*. *agadiriana* and the *barbata* species, as revealed by cytological studies [45] and by molecular data [9, 13, 14]. In the current study, two distinct types of *Pgk1* sequences were obtained in *A*. *agadiriana*. One copy clustered with the A_c_ genome species *A*. *canariensis*, whereas the other copy fell into cluster A2 with the A_s_ species *A*. *atlantica*, *A*. *hirtula*, *A*. *lusitanica*, and the hexaploids *A*. *occidentalis*, *A*. *fatua* and *A*. *sativa* (Fig 3 and S1 Fig). These results were in agreement with our previous studies based on nuclear *Acc1* gene [14] and GBS markers [9], and they support the proposal that *A*. *agadiriana* contains a different combination of A and/or B genomes from the *barbata* group, and that one of its two genomes originates from the A_c_ genome species *A*. *canariensis*, whereas the other one is closely related to the A_s_ species.

### The tetraploid species *A*. *maroccana* and *A*. *murphyi* are closely related to the hexaploids, while *A*. *insularis* is diverged

The other tetraploid group (*Avena* sect. *Pachycarpa*) contains three species, *A*. *maroccana*, *A*. *murphyi*, and the recently discovered *A*. *insularis*. Initial studies based on genomic in situ hybridization [46] supported an AC genome designation for these species. However, this designation has been challenged by FISH analysis, which has revealed that this set of tetraploid species, like the D chromosomes of the hexaploid oats, lacks a repetitive element that is diagnostic of the A genome [18]. This, together with other molecular evidence [14, 47] and our recent whole-genome analysis based on GBS markers [9], suggests that these tetraploid species contain the genome designated as D in hexaploid oats, and that they are more properly designated as DC genome species.

In the present study, two distinct *Pgk1* homoeologues were detected in each of the three AC(DC) species, with each pair falling consistently into two clusters within the C and the A genome clades, respectively (Fig 3 and S1 Fig). The C-copy sequences of these tetraploids clustered consistently with the C-type homoeologues of the hexaploids, while the A/D genome homoeologues, with the exception of these from *A*. *insularis* and one sequence from *A*. *maroccana* (CIav 8331) fell into subclade A3 along with a set of sequences from the hexaploid oats (Fig 3). Considering that the other *Pgk1* gene sequences from the hexaploid oats clustered with the C or A genome diploids, we deduced that the sequences falling in subclade A3 must represent the D genome homoeologues of the hexaploids and of the AC(DC) species *A*. *maroccana* and *A*. *murphyi*. This result is not fully consistent with our previous GBS study: although *A*. *maroccana* and *A*. *murphyi* were very similar to hexaploid oat and were designated as DC genomes, our GBS work suggested that *A*. *insularis* was also a DC genome that was even more similar to the hexaploids [9]. Examining the existing literature, all three of these tetraploid species have variously been considered as the tetraploid ancestor of the hexaploids [4, 9, 48]. In view of the genome structure of these tetraploids [24, 49] and the meiotic chromosome paring of their interspecific hybrids [50], all of these tetraploids are proposed to have diverged from a common ancestral tetraploid after the occurrence of some large chromosome rearrangements [24, 49]. However, it cannot be ruled out that these tetraploids might have originated independently from different diploid ancestors, since they have shown close relationships with different diploid species [40]. Interestingly, the A/D-type homoeologues of *A*. *insularis* fell into a group with the A_c_ genome species *A*. *canariensis* and the AB genome species *A*. *agadiriana*. In fact, previous studies have revealed that *A*. *canariensis* is closely related to the DC genome tetraploids [15]. These results suggest a possibility that *A*. *canariensis* was involved in contributing an early version of a D genome in all three AC(DC) genome tetraploids. Nevertheless, we do not have an explanation for why the D genome copy of *Pgk1* in *A*. *insularis* could have diverged so far from the version found in the hexaploids, especially since the C genome copies remain identical.

### The genome origins of the hexaploid species

It is now generally accepted that two distinct steps were involved in the evolution of hexaploid oats. The first step would have been the formation of a DC genome hybrid from ancestral D and C genome diploids, followed by doubling of the chromosomes to form an allotetraploid. The second step would have involved hybridization of a DC tetraploid with a more recent A genome diploid, followed by doubling of the triploid hybrid [9, 13].

The diploid progenitor of the hexaploid C genome was probably restricted to the narrow geographic range where the three extant C genome diploids are distributed. However, numerous inter-genomic translocations among hexaploid chromosomes [9, 11, 51, 52] have decreased the homology between the C genome diploids and the hexaploid C genome, making the identification of the C genome donor of the hexaploids challenging. In this study, the C_p_ genome species shared the highest degree of genetic similarity with both the AC(DC) genome tetraploids, as well as with the hexaploids, leading us to conclude that a C_p_ genome species was the C genome donor of the polyploids. This conclusion is supported by other evidence from nuclear genes [13, 53]. This is important, since it was recently demonstrated that the maternal tetraploid and hexaploid genomes originated from an A genome species, not from a C genome species [54], rendering comparisons to the C_v_ *vs* C_p_ maternal genomes irrelevant in determining the origin of the nuclear C genome in the hexaploids.

The A genome origin of the hexaploids remains a matter of debate, and many A genome diploids have been suggested as putative diploid progenitors, as summarized by Peng et al. [13]. FISH analysis showed that an A_s_-specific DNA repeat was restricted to the A_s_ and A_l_ genomes, as well as the hexaploid A genome [18]. In this study, a close relationship between the A_s_ genome diploid *A*. *atlantica* was observed for some hexaploid haplotypes in the phylogenetic tree (Fig 3) and the MJ network (Fig 4). An *A*. *atlantica* genome origin is consistent with previous studies based on IGS-RFLP analysis [12] and the *ppcB1* gene [40]. However, there is evidence in our work that some hexaploids may have an alternate A genome origin, closer to the *Agraria* section of A_s_ diploids. The presence of multiple A genome origins could explain variable results that have been reported in studies of hexaploid phylogeny.

In this study, strong evidence is presented for a D genome origin in the tetraploids *A*. *maroccana* and *A*. *murphyi* (Figs 3 and 4). However, these D genome sequences did not show a close relationship with any diploid species investigated in this study. Other than the discrepancy with *A*. *insularis*, this result is consistent with our recent GBS study [9]. One factor that may hinder the discovery of a D genome progenitor is the presence of inter-genomic translations among all three genomes in the hexaploid [9, 52]. Two hexaploid accessions (*A*. *occidentalis* CN 25942 and *A*. *sativa* PI 194896) did not contribute haplotypes that clustered with the putative D genome sequences (Subclade A3 in Fig 3). Although this may be a result of incomplete sampling, it may also result from inter-genomic translations that have duplicated or eliminated copies of *Pgk1*.

In conclusion, this is the most comprehensive study to date that investigates a phylogeny in genus *Avena* using a single informative nuclear gene. It confirms or clarifies most previous work, and presents strong evidence in support of a working hypothesis for the origin of hexaploid oat. However, many questions still remain, and these will be best addressed through further studies involving multiple nuclear genes or whole genomes. We are collaborating on work that will provide exome-based gene diversity studies, but this work will require complete hexaploid reference sequences before it can be properly analyzed. Such reference sequences are currently in progress, so the next few years may see a revolution in our understanding of *Avena* phylogeny. Nevertheless, as many researcher in this field are aware, the polyploid species in this genus have experienced extensive chromosome rearrangement, which will continue to complicate phylogenetic studies. It may even be necessary to generate a pan-genome hexaploid reference sequence before definitive statements can be made.

## Supporting information

S1 Fig Consensus tree based on 110 *Pgk1* sequences reconstructed using Bayes inference. The GTR+Γ+I model was chosen as the best-fit substitution model by using MrModelTest v2.3 under AIC. Bayesian posterior probability (PP) values equal or more than 90% are showed above the branches. Accession number, species name and haplome are indicated for each taxon.
(TIF)
