# Supplementary figures and images for: Phylogenetic relationships in the genus Avena based on the nuclear Pgk1 gene
Source: PLoS One. 2018 Nov 8;13(11):e0200047. doi: 10.1371/journal.pone.0200047 (PMC6224039; doi:10.1371/journal.pone.0200047)

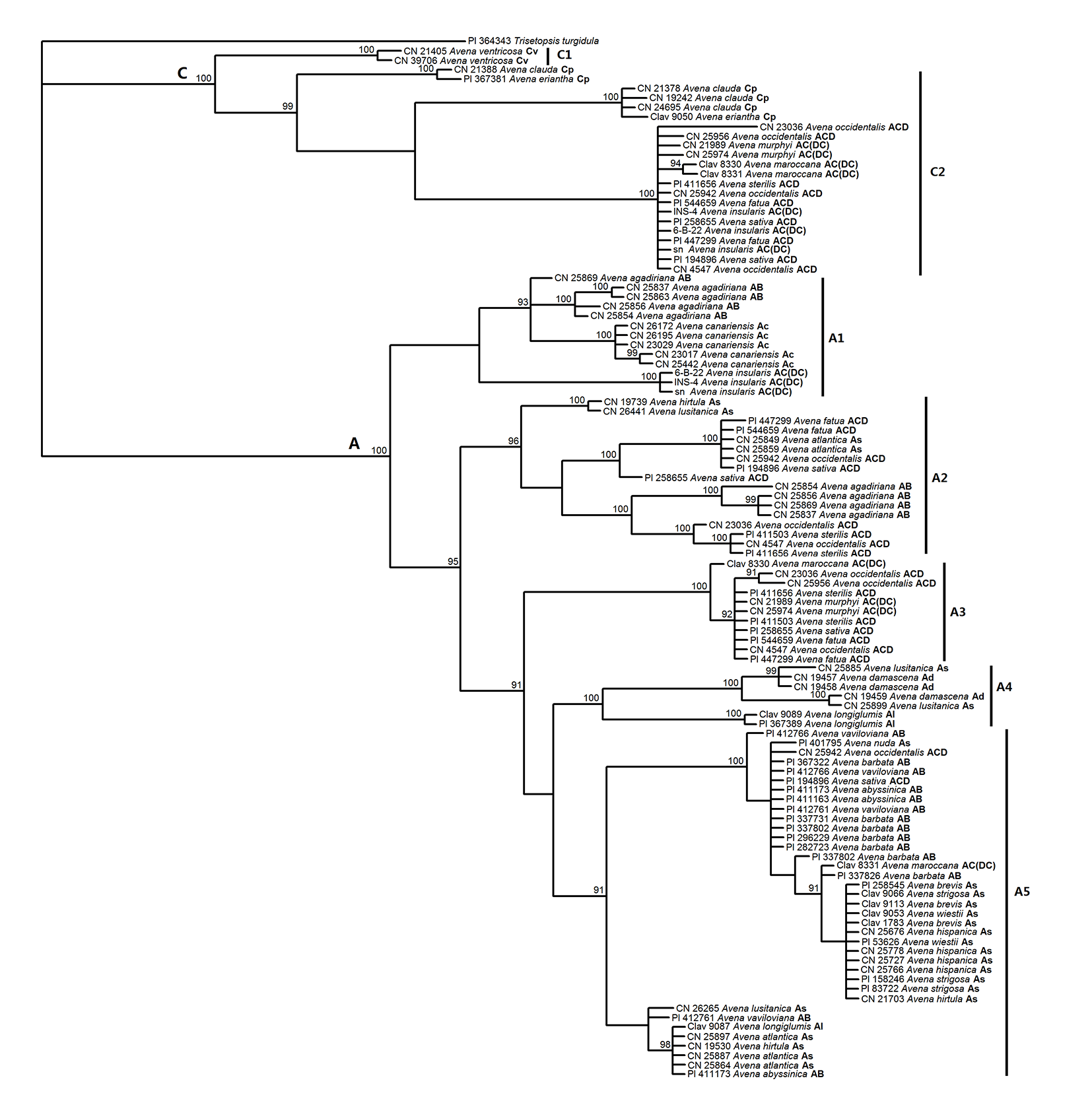

Supplement: S1 Fig — The GTR+Γ+I model was chosen as the best-fit substitution model by using MrModelTest v2.3 under AIC. Bayesian posterior probability (PP) values equal or more than 90% are showed above the branches. Accession number, species name and haplome are indicated for each taxon. (TIF) [file pone.0200047.s001.tif]
